# The next step: mirror neurons, music, and mechanistic explanation

**DOI:** 10.3389/fpsyg.2015.00409

**Published:** 2015-04-14

**Authors:** Jakub R. Matyja

**Affiliations:** ^1^Philosophy, Polish Academy of SciencesWarsaw, Poland

**Keywords:** mirror neurons, embodied music cognition, mechanistic explanation, music cognition, psychology of music

## Introduction

The next step for the paradigm of embodied music cognition (EMC) is to move from building theories of (empirical) data toward building systematic explanations of organisms' capacities to process music. I argue that for the mirror neuron (MN) hypothesis to be effective when applied to music, these neurons' capacities should be proven to be nontrivially and causally connected with musical organisms as a whole. This requires, I argue, a mechanistic type of explanation.

## Mirror neurons and music

The reception of the MN hypothesis within EMC has been warm at least since the start of systematic research in this paradigm (Leman, 2008). Researchers working on mirror neurons (e.g., Jeannerod, 2001) speculated that since these neurons are activated during the observation and the execution of a monkey's motor actions, they enable the animal to understand others' behavior by simulating their actions in its own brain. Although the non-human primates themselves are not musical (McDermott and Hauser, 2007), the MN hypothesis was quickly adopted in musical research (Molnar-Szakacs and Overy, 2006). Even if the exact counterparts of mirror neurons have not been yet found in the human brain, the EMC research community (Matyja and Schiavio, 2013) has approached the very idea of them internally simulating the musical behaviors of others with huge enthusiasm. For instance, the analogs of the MN system in humans are now hypothesized to be connected with (or at least in some way an explanatory factor in) understanding facial expressions and nonverbal communication in musical ensembles (Vines et al., 2006), embodied simulation of music (Schiavio et al., 2015), distinguishing self from others (Novembre et al., 2012), and enabling the understanding of intentions embedded in music (Corness, 2008). All of the above-mentioned tasks require MNs to internally simulate (musical) actions or the intentions of others or the content conveyed by a given musical signal.

## Embodied simulation and music

Overy and Molnar-Szakacs (2009), being perhaps the most influential researchers working on MN and music declare that MN *may* serve as a basis for providing models of phenomena that are of direct interest to the EMC community. Yet we still do not have any idea of *how they may do it*. Current references to the operation of human analogs of the MN system's *activations* rely on the black box type of explanations, in which they are supposed to play an important but vaguely specified role in producing the behavioral output given the perceptual input. Consider the following example: Cochrane (2010, p. 20) provides what he has dubbed the “simulation theory of musical expressivity,” in which “music is seen as hijacking the simulation mechanism of the brain.” Cochrane's argument is based on three main steps. The causal process introduced there goes as follows. First, there is a (a) the triggering of a brain's emotion-detecting simulation mechanism, which is done either by belief or imagination of the agency generating the sound. Secondly, (b) the intermodal connection between sound and bodily movements is utilized, which then leads to (c) the mirroring of these movements from a first-person perspective, which elicits a simulation of emotions in the listener (Schiavio et al., 2015). However, the crucial problem here is that we have no idea how MNs do it. Cochrane's theory leaves the answer to this particular question hanging upon the further theoretical clarifications and/or empirical confirmations of whether MNs themselves are sufficient for systematically explaining the process of simulation. For these two reasons, we now need to advance to the next step.

## Next step: “how” mirror neurons do it?

Our current state of knowledge about activations of the MN system counterparts in humans relies upon the heuristics of localization (Bechtel and Richardson, 1993) common to neuroscientific data collection methods. By localization here, I simply refer to the identification of the component parts (e.g., activation of motor cortices) assumed to be causally involved in the execution of given tasks. This scientific strategy is may fail to be explanatory (Weisberg et al., 2008; Carp, 2012), and does not answer the question how the phenomena in question occur. Following that thought, from a mechanistic explanation's perspective the individual parts of an organism (e.g., motor cortices of the brain) do not themselves realize the working of a given mechanism that produces the phenomenon to be explained. There is a long way from observing the activation of the human counterparts of MNs system to explaining the phenomenon of musical simulation. Systematic explanation of the human capacity for musical simulation requires a lot more than the sheer localization of the neural components within the boundaries of the skull. Because of the commitments of embodied approaches to music cognition (Matyja and Schiavio, 2013), we should account for how the whole body shapes the ways in which we simulate music. While the research on the neural correlates is important, the systematic explanation of the human capacity for musical simulation requires decomposition. I assume that the embodied cognitive system responsible for the phenomenon of musical simulation is hierarchical and decomposable. In order to see how musical simulation results from different parts and their activities—following the mechanistic explanation strategy (Bechtel and Richardson, 1993) we must actually decompose the phenomenon into the component operations that produced it and localize them within the parts of the embodied cognitive mechanism.

## Mechanistic explanation

Mechanistic explanations typically proceed in three steps. We begin by identifying the phenomenon to be explained, the *explanandum phenomenon*. Secondly, we focus on the phenomenon's decomposition into a number of entities and activities that are relevant to the explanation. Finally, mechanistic explanations account for these entities and activities, driven by the question of how they are organized in order to be able to produce the given phenomenon (Illari and Williamson, 2012). For the mechanism to be adequately described, we need to account for the relations within and between the bottom (neural), the isolated (embodied), and the contexual (situated) levels (Bechtel, 2009) on which we analyze music simulation. To illustrate this, take an biological example of the reproduction of yeasts. The process of reproduction via budding takes place in a given environment (contextual level). It is explained in terms of division (occurring on the isolated level that ignores the environment) as well as division in terms of cellular mechanisms (which is in this example the bottom level, possibly explained further, if needed, by its molecular parts). Now consider how (cognitive) scientists build models. They usually start with a hypothesis of how something works. In our case, the members of the EMC research community typically assume that MNs are responsible for various simulations. On the basis of this initial hypothesis, they build a model. From a mechanistic explanation's perspective, which is an account of the components and operations that are necessary to produce an occurrence of the studied phenomenon. Take, for instance, the successful mechanistic explanation of visual processing (as reported by Bechtel, 2009). While the non-mechanistic accounts often decompose (and, inherently reduce) visual processing into “parts” operating in sequences, Bechtel reminds us of the importance of taking the next step: recomposing the investigated mechanism. This move is performed taking into account the organization among these parts, as well as *situating* the mechanism in its environments.

## Mechanism sketch for mirror neurons: a primer

In my opinion, the goal of achieving a systematic explanation of how MNs work requires understanding of how a whole organism involved in musical interaction. Accordingly, the cases like musical simulation (in Cochrane's sense) ought to be accounted for in terms of three interrelated levels of explanation derived through analysis: bottom, isolated, and contextual. The first—bottom (neural) level encompasses the single neuron and neural patterns activations. The second—isolated (embodied) level—is at least partially concerned with drawing broader and often wild conclusions from the available empirical data. The primary explanatory heuristic used in these analyses is “Inference to the Best Explanation” (Okasha, 2000). The idea of this heuristic is that a given hypothesis should be inferrable from the available evidence. However, the fact that MN system research is currently under critique (Kilner et al., 2003; Hickok, 2014) suggests that one should be, at least, cautious in interpreting the role of their activations (Shapiro, 2011, p. 111; for a mechanistic explanation of the workings of the MN system, see: Herschbach, 2012). From the mechanistic perspective, the idea behind the isolated level is to examine how a given mechanism works as itself, isolated from its environment and without implicating lower-level structures and functions. It is thus consistent with laboratory studies that include stimulation and recording spike trains from an isolated neuron or studying a human subject's responses to computer-generated stimuli, which are both examples of this strategy (Wright and Bechtel, 2007). Finally, the contextual level. As argued by numerous researchers working in the EMC paradigm, our musical interactions are not only subject to bodily-environmental interactions, but are also situated within historical and cultural constraints (Clarke, 2005; Leman, 2008). These intuitions are (at least) consistent with mechanistic explanations. For instance Bechtel (2009) underlines the high risk of underestimating the significance of environmental structures, therefore pointing to the critical importance of experimentation in organisms' natural environments.

## Conclusions

In my opinion, the efficacy of the MN hypothesis as applied to music cognition relies crucially on providing a mechanistic explanation of how these neurons interact with the components of an organism involved in musical interactions. While we currently assume that the role of MNs in music cognition is to *simulate*, EMC researchers should start accounting for how these neurons do it exactly. This next step, which essentially requires a shift from relying on the neuroscientific heuristic of localization (i.e., of the human activations counterpart of MN) to the heuristics of decomposition (i.e., understanding how they interact with embodied organisms as whole) and further recomposition of the identified mechanism via situating it in its (both bodily and musical) environment. The next step for the EMC research paradigm is thus to consider the value of mechanistic explanations.

### Conflict of interest statement

The author declares that the research was conducted in the absence of any commercial or financial relationships that could be construed as a potential conflict of interest.
